# Redefining Maternal Wellness: The Role of Antenatal Exercises in Musculoskeletal Issues Among Primigravida Mothers

**DOI:** 10.7759/cureus.50494

**Published:** 2023-12-14

**Authors:** Ambika K, Vijayalakshmi V

**Affiliations:** 1 Obstetrics and Gynecological Nursing, Government Medical College Hospital, Chennai, IND; 2 Obstetrics and Gynecological Nursing, College of Nursing, Madras Medical College, The Tamilnadu Dr. M.G.R. Medical University, Chennai, IND

**Keywords:** pregnancy-related ailments, maternal wellness, musculoskeletal ailments, primigravida mothers, antenatal exercises

## Abstract

During pregnancy, there are notable alterations in biomechanics, hormones, and vascular functioning, which frequently result in a range of musculoskeletal ailments, including back pain, leg cramps, and pelvic girdle discomfort. The significance of pregnancy-related musculoskeletal problems on women's daily functioning and general well-being is highlighted by their widespread occurrence worldwide, necessitating heightened focus and implementation of effective therapeutic approaches. The main aims of this study were to assess the effectiveness of prenatal exercises in musculoskeletal discomfort and investigate the association between post-intervention levels of discomfort and certain demographic factors. A quantitative technique was used in this study, utilizing a pre-experimental design conducted for three months. A total of 60 primigravida mothers were selected as participants through purposive sampling. The study was conducted in a Maternity Tertiary Care Center located in Tamil Nadu. The intervention encompassed the provision of antenatal exercises, specifically focusing on abdominal tightness, pelvic tilting, and foot and ankle movements. The researcher demonstrated the exercises for 20 minutes, and afterward, mothers were asked to perform the activities themselves. The process was monitored and observed for two weeks. The findings were statistically significant, suggesting a noteworthy decrease in musculoskeletal disorders following the implementation of the intervention. The statistical analysis revealed a significant degree of significance (*P *= 0.001), confirming the efficacy of the exercises. Before the implementation of the intervention, a significant proportion of mothers, namely, 45 (75%) reported experiencing moderate back pain. However, following the intervention, this percentage notably fell to 33.34% (20). The incidence of moderate pelvic pain decreased from 80% (48) to 30% (18), and a comparable pattern was observed in the reduction of leg cramps. Additionally, the research identified significant associations between the improvements and a range of demographic and obstetric factors, including the level of education, occupation, family structure, age at marriage, and weight of the mother. The results highlight the significance of incorporating antenatal exercises as a regular component of prenatal care to minimize musculoskeletal discomfort, hence promoting the overall health and well-being of expectant mothers.

## Introduction

Pregnancy represents a significant period of transformation in a woman's life, characterized by notable manifestations of creative and nurturing capacities [1]. This is a critical phase during which maternal health significantly impacts the overall welfare of the developing fetus. This particular period is distinguished by notable physical and physiological transformations, as the human body adjusts to facilitate the development of the growing fetus within the uterus [2]. The physiological alterations in biomechanics, hormone regulation, and vascular dynamics that occur during pregnancy have been associated with a diverse array of musculoskeletal problems. The displacement of the uterus during pregnancy results in a redistribution of the body's center of gravity, hence imposing mechanical strain on the physiological system [3]. Hormonal variations during pregnancy contribute to joint laxity, while fluid retention can exert pressure on soft tissues, rendering pregnant women more vulnerable to musculoskeletal problems. Frequently reported issues encompass a range of common ailments, such as back pain, leg cramps, and peripheral neuropathies, with spinal pain being the predominant concern [4].

The occurrence of pregnancy-induced neuromechanical changes, including modifications in stride, posture, and sensory input, escalates the susceptibility to musculoskeletal problems and fall-related accidents [5]. As an example, the pelvis undergoes a tilting motion, causing the back to arch to sustain equilibrium, frequently resulting in suboptimal postural alignment. Moreover, the progressive increase in body mass and hormonal fluctuations experienced during pregnancy can have an impact on the foot, contributing to feelings of pain [6]. Recent research has indicated that musculoskeletal disorders exhibit the highest prevalence during the second and third trimesters of pregnancy. In the absence of appropriate therapy, these relatively mild disorders have the potential to intensify, thereby impacting the well-being of both the expectant mother and the developing fetus [3]. The dissemination of information regarding these matters to expectant mothers is of utmost importance, as it necessitates no specialized apparatus, but rather relies on the presence of a competent midwife educator and the receptiveness of the mothers to engage in attentive listening and adherence to instructions actively [7].

The global burden of musculoskeletal issues during pregnancy has been highlighted by the World Health Organization (WHO), prompting the organization to organize meetings aimed at enhancing rehabilitation services for these illnesses [8]. The incidence of pregnancy-related low back pain and pelvic girdle discomfort exhibits substantial global variation, exerting a notable impact on individuals' daily functioning and overall well-being. Within the Indian context, much research has been conducted to investigate the prevalence and consequential effects of musculoskeletal discomfort experienced during pregnancy [9]. A study conducted in Tamil Nadu revealed that a significant proportion of pregnant women encounter various physical discomforts, particularly during the advanced stages of pregnancy [10,11]. The researchers at the Institute of Obstetrics and Gynaecology in Chennai discovered that a notable percentage of primigravida women encounter musculoskeletal issues in the latter stages of pregnancy, specifically during the second and third trimesters.

Developing comprehensive preventative and treatment strategies can be facilitated by gaining an understanding of the typical discomforts experienced during different trimesters [12,13]. Numerous studies conducted both in India and internationally have underscored the necessity of implementing such efforts. The authors propose that musculoskeletal discomforts, including lower back and hip pain, are widespread and have a substantial negative impact on the overall quality of life experienced by pregnant individuals [14,15]. The recognition of prenatal exercises in the management of various diseases is growing. These exercises have the objective of improving the overall physical and psychological health of pregnant women and reducing the occurrence of pregnancy-related disorders [16,17]. Typically, prenatal exercise routines consist of low-impact aerobic workouts and stretching, which are characterized by their ease of execution and ability to effectively alleviate discomfort and minimize the duration of childbirth preparation.

Nevertheless, there exists a deficiency in understanding antenatal exercises, particularly among first-time pregnant women [18]. The objective of this study is to evaluate the efficacy of antenatal exercises in mitigating musculoskeletal disorders among primigravida women who are receiving care at an antenatal outpatient facility. This study aims to fill a gap in current knowledge and practice by examining the effects of prenatal exercises on the well-being of pregnant women, offering valuable insights into this area.

## Materials and methods

The primary objective of the research study was to assess the efficacy of antenatal exercises in musculoskeletal discomfort experienced by primigravida mothers and to associate them with selected sociodemographic variables. The study used a quantitative methodology, specifically utilizing a pre-experimental design known as the one-group pretest-posttest design. The antenatal exercises consisted of three components: abdominal tightness, pelvic tilting, and foot and ankle exercises, which served as the independent variable in the study. The variable of interest in this study was the occurrence and intensity of musculoskeletal ailments among first-time pregnant mothers. 

The research was carried out for three months at the antenatal outpatient department of the Maternity Tertiary Care Center located in Tamil Nadu. The study focused on primigravida mothers who were in their second and third trimesters and were attending the antenatal outpatient department. The researchers utilized a purposive sampling method, selecting a sample of 60 primigravida mothers who met certain inclusion criteria. These criteria included a willingness to participate, proficiency in either Tamil or English, and the presence of only mild to moderate musculoskeletal complaints. The exclusion criteria encompassed individuals with mental disability, high-risk medical disorders, prior antenatal exercise experience, severe musculoskeletal diseases, and utilization of pain treatment techniques. The instrument utilized for the collection of data was a pain scale that was designed and verified using input from medical professionals and authorities in the nursing department. The study incorporated demographic and obstetric factors, as well as a numerical pain rating scale that spanned from 0 (indicating the absence of pain) to 10 (representing intense pain). The reliability of the tool was validated through the attainment of a strong correlation coefficient of 0.92.

The ethical aspects of the study were comprehensively handled, as evidenced by the permission received from the Institutional Ethics Committee of Madras Medical College and the acquisition of informed consent from all mothers involved. The pilot study, which included a sample size of 10 mothers, provided evidence to support the practicality of the primary study. The process of data collecting encompassed several key steps, including establishing initial contact with participants, obtaining informed consent, and conducting a pre-assessment utilizing the numerical pain scale. The researcher demonstrated the exercises for 20 minutes, after which the mothers were asked to perform the activities themselves. The process was monitored and observed for two weeks. Subsequently, antenatal exercises were illustrated, followed by a post-assessment that took place two weeks later. The intervention protocol outlined the specific details of the location, exercises, duration, teaching approach, and posttest evaluation. The statistical techniques used in this study include descriptive statistics (frequency distributions, percentages, and mean ), inferential statistics (chi-square test and extended McNemar's test)

## Results

Sociodemographic and obstetric variables

The study examined 60 first-time mothers (Table 1), most of whom were aged between 21 and 30 years (49, 81.67%). Twenty-six (43.33%) mothers had been married for one to two years, while the majority of them were in their first one to three years of marriage. The average level of education varied, with secondary education being the most common (31, 51.67%) and primary education being the next most common (23, 38.33%). Of the total number of participants, 49 (81.66%) were Hindu and 45 (75%) were homemakers. The majority of them (51, 85%) earned between Rs. 10,001 and Rs. 12,000 a month, and most of them lived in cities (34, 56.67%). The majority of the participants got married between the ages of 18 and 24 years.

**Table 1:** Distribution of demographic variables of the study population.

Serial no.	Demographic variables		Primigravida mothers (*n*)	%
					1	Age	18-20 years	7	11.67
21-30 years	49	81.67				
>30 years	4	6.66				
2	Duration of marriage	Less than 1 year	5	8.33
		1-2 years	26	43.33
		2-3 years	25	41.67
		3 years and above	4	6.67
3	Education	Nonliterate	6	10.00
		Primary education	23	38.33
		Secondary education	31	51.67
		Graduate and above	0	0.00
4	Religion	Hindu	49	81.67
		Christian	5	8.33
		Muslim	6	10.00
5	Occupation	Homemaker	45	75.00
		Skilled worker	5	8.33
		Sedentary worker	10	16.67
		Self-employed	0	0.00
6	Monthly income	Rs.	9	15.00
		Rs. 10,001-20,000	51	85.00
		Rs. 20,001-30,000	0	0.00
		Rs. 30,001	0	0.00
7	Place of residence	Urban	34	56.67
		Semi-urban	6	10.00
		Rural	20	33.33
		Slum	0	0.00
8	Age at marriage (in years)	18-20	23	38.33
		21-24	24	40.00
		25-30	13	21.67
9	Type of family	Nuclear family	30	50.00
		Joint family	22	36.67
		Extended family	8	13.33
10	Type of marriage	Consanguineous	5	8.33
		Non-consanguineous	55	91.67

According to the obstetric data presented in Table 2, the majority of mothers had a gestational age ranging from 29 to 32 weeks, accounting for 35 (56.32%) participants in this study. The distribution of antenatal checkups was evenly split between government hospitals and primary health centers, with each accounting for 46.67% (28) of the total. The majority of participants (42, 70%) reported attending three antenatal visits. The majority of heights fell within the range of 151-160 cm, accounting for 32 (53.33%) samples. Similarly, the most prevalent weight category was 51-60 kg, representing 34 (56.67%) participants. All participants were enrolled in the Perinatal and Infant Care Monitoring and Evaluation (PICME) system and received immunizations. No prior medical or obstetric complications were documented.

**Table 2:** Obstetric information among primigravida mothers.

Serial no.	Obstetric variables		Primigravida mothers, *n*	%
1	Gestational age (in weeks)	24-28	25	41.67
		29-32	35	56.32
		32-36	4	2.01
2	Place of antenatal checkup	Government hospital	28	46.67
		Private hospital	4	6.67
		Primary health center	28	46.66
3	Number of antenatal visits	One	0	0.00
		Two	6	10.00
		Three	42	70.00
		More than three	12	20.00
4	Height (cm)	140-150	17	28.33
		151-160	32	53.33
		More than 160	11	18.33
5	Weight (kg)	30 -40	0	0.00
		41-50	15	25.00
		51-60	34	56.67
		More than 60	11	18.33
6	Pregnancy and Infant Cohort Monitoring and Evaluation (PICME) Registration	Yes	60	100.00
		No	0	0.00
7	Are you immunized?	Yes	60	100.00
		No	0	0.00
8.	Do you have any previous medical, obstetric, or surgical problems?	Yes	0	0.00
		No	60	100.00

Pre-intervention levels of musculoskeletal ailments

Before the implementation of the antenatal exercise intervention, there was a notable prevalence of moderate musculoskeletal pain observed among the maternal population. Forty-five (75%) participants reported experiencing moderate back pain, while 48 (80%) reported pelvic pain and 47 (78.33%) reported leg cramps. The observed high prevalence of moderate pain levels suggests a significant burden of musculoskeletal disorders within the population under investigation (as evidenced by the data presented in Tables 3-5).

**Table 3:** Pretest level of back pain score among primigravida mothers.

Level of score	Number of primigravida mothers, *n*	Total percentage (%)
Mild pain	15	25.00
Moderate pain	45	75.00
Total	60	100.00

**Table 4:** Pretest level of pelvic pain score among primigravida mothers.

Level of score	Number of primigravida mothers, *n*	Total percentage (%)
Mild pain	12	20.00
Moderate pain	48	80.00
Total	60	100.00

**Table 5:** Pretest level of leg cramp scores among primigravida mothers.

Level of score	Number of primigravida mothers, *n*	Total percentage (%)
Mild pain	13	21.67
Moderate pain	47	78.33
Total	60	100.00

Effectiveness of antenatal exercises

The implementation of antenatal exercises demonstrated a significant decrease in musculoskeletal ailments. Following the session, a notable decrease in back pain was seen, with 40 (66.66%) participants reporting light discomfort. This reduction was statistically significant compared to the participants' pre-intervention condition. In a similar vein, the prevalence of mild pelvic pain rose to 42 (70%) following the intervention, while 44 (73.33%) mothers reported experiencing mild leg cramps. The findings of this study highlight the efficacy of antenatal exercises in reducing the intensity of musculoskeletal pain experienced by first-time pregnant mothers (Tables 6-8).

**Table 6:** Comparison of pretest and posttest levels of back pain scores among primigravida mothers.

Level of score	Pretest		Posttest		Extended McNemar’s test
	n	%	n	%	
Mild	15	25.00	40	66.66	Chi-square value* *= 28.30; *P*-value = 0.001 (significant)
Moderate	45	75.00	20	33.34	
Total	60	100.00	60	100.00	

**Table 7:** Comparison of pretest and posttest levels of pelvic pain scores.

Level of score	Pretest		Posttest		Extended McNemar’s test
	n	%	n	%	
Mild	12	20.00	42	70.00	Chi-square value = 32.50; *P*-value = 0.001 (significant)
Moderate	48	80.00	18	30.00	
Total	60	100.00	60	100.00	

**Table 8:** Comparison of pretest and posttest levels of leg cramp pain scores.

Level of score	Pretest		Posttest		Extended McNemar’s test
	n	%	n	%	
Mild	13	21.67	44	73.33	Chi-square value = 34.40; *P*-value = 0.001 (significant)
Moderate	47	78.33	16	26.67	
Total	60	100.00	60	100.00	

Association with sociodemographic variables

The pain levels observed after the intervention had different associations with sociodemographic characteristics. The results of the study indicate that individuals who identified as educated women (*P *= 0.05) and homemakers (*P *= 0.01) saw a more substantial reduction in pain levels following the intervention. This finding suggests that the lifestyle of homemakers may have played a beneficial role in enhancing the efficiency of the exercises. There was a discernible association observed between the kind of family structure and the extent of reported improvements, with nuclear families (*P *= 0.01) exhibiting more substantial progress. This phenomenon may be attributed to the presence of enhanced support systems or the provision of more individualized care within smaller family groups. Regarding obstetric characteristics, there was a notable association observed between the age at marriage (*P *= 0.01) and weight of mothers (*P *= 0.05) and the levels of pain experienced. Mothers with a body weight over 60 kg reported higher levels of moderate pain scores following the intervention, suggesting that the efficacy of prenatal workouts may be influenced by body weight.

## Discussion

The findings of the study offer significant insights into the effects of prenatal activities on musculoskeletal ailments in first-time pregnant mothers. The initial findings of the study indicated a significant incidence of musculoskeletal disorders among primigravida mothers, with a majority reporting moderate levels of back pain, pelvic discomfort, and leg cramps. The results align with the research conducted by Onyemaechi et al., wherein a similar pattern of elevated prevalence of musculoskeletal impairments, such as calf muscle cramps and low back pain, was observed among pregnant women, particularly during the advanced stages of pregnancy [19]. The findings from both studies highlight the escalating physical strain experienced during pregnancy, as seen by the heightened symptomatology observed in each successive trimester.

The considerable reduction in pain levels post-intervention indicates the effectiveness of antenatal exercises. This correlates with the findings of Davenport et al., where a specialized exercise program resulted in lower pain intensity and better functional abilities in pregnant women with low back pain [20]. Similarly, the research by Stuge indicated that pelvic girdle exercises considerably reduced pelvic girdle pain and enhanced specific tasks. The unifying thread in this research is the emphasis on targeted exercises to treat specific musculoskeletal disorders, underlining the usefulness of such interventions in prenatal care [21].

The study also evaluated the association of pain reduction with demographic and obstetric factors. Interestingly, educated mothers, homemakers, younger mothers, and those from nuclear households exhibited more significant benefits. This shows that lifestyle characteristics, familial support systems, and age may play roles in how efficiently pregnant women can manage and lessen musculoskeletal discomfort. Additionally, the result echoes the research by Fiat et al., which indicated physical inactivity and body weight to be major factors influencing musculoskeletal pain during pregnancy. This confirms the view that a holistic approach, encompassing lifestyle and physical health, is vital in managing pregnancy-related ailments [22].

The results underline the crucial role of nurses in prenatal care, particularly in teaching and encouraging primigravida mothers to undertake antenatal exercises. Nurses can function as catalysts in promoting these activities, highlighting their benefits not just in lowering musculoskeletal illnesses but also in enhancing the general quality of life during pregnancy. This study, through its findings and comparisons with current literature, underlines the need for integrating exercise regimens into normal antenatal care, customized to the unique needs of pregnant women. While the study offers valuable information, further research might address the long-term impact of antenatal activities beyond immediate pain reduction, including postpartum healing and mental well-being. The limitation of the current study is its focus on a specific cohort (primigravida mothers), which may not be generalizable to all pregnant women. Further research should widen the demographic reach to include multigravida women and study diverse geographical and cultural situations to validate these findings more extensively.

## Conclusions

The study assessing the efficacy of antenatal exercises in alleviating musculoskeletal disorders among first-time pregnant women provides valuable insights into prenatal treatment strategies. The research findings provide clear evidence that implementing certain antenatal exercises effectively reduces the occurrence and intensity of musculoskeletal ailments, including back pain, pelvic discomfort, and leg cramps, in first-time pregnant women. The significant decrease in pain levels following the intervention highlights the need to integrate regular exercise regimens into prenatal care. The results of this study are supported by previous research, providing additional evidence for the significant impact of physical activity on the overall health and wellness of pregnant women. The findings of this study are of great significance not only for the field of clinical practice but also for informing the development of effective strategies for prenatal care.

Additionally, the study emphasizes the crucial involvement of nurses and healthcare professionals in advocating for and implementing these exercise routines. By incorporating antenatal exercises into standard prenatal care and providing personalized coaching to pregnant women, healthcare practitioners have the potential to greatly enhance the well-being of expectant mothers. This methodology has the potential to yield improved health outcomes for both the maternal and their children. The results of the study also suggest the need for additional research in this field, particularly about varied populations and the long-term impacts of prenatal activities. The study serves to confirm the fundamental significance of engaging in physical activity during pregnancy and emphasizes the necessity for its wider implementation in prenatal healthcare.
